# The activity of methylene blue against asexual and sexual stages of *Plasmodium vivax*

**DOI:** 10.3389/fcimb.2023.1108366

**Published:** 2023-04-18

**Authors:** Camila Fabbri, Glenda Quaresma Ramos, Djane Clarys Baia-da-Silva, Alexandre Oliveira Trindade, Luis Carlos Salazar-Alvarez, Juliana Costa Ferreira Neves, Ivanildes dos Santos Bastos, Allyson Guimarães Costa, Marcus Vinicius Guimarães Lacerda, Wuelton Marcelo Monteiro, Fabio Trindade Maranhão Costa, Stefanie Costa Pinto Lopes

**Affiliations:** ^1^ Instituto Leônidas & Maria Deane, Fiocruz Amazônia, Manaus, Brazil; ^2^ Fundação de Medicina Tropical Dr. Heitor Vieira Dourado, Manaus, Brazil; ^3^ Escola Superior de Ciências da Saúde, Centro Multiusuário para Análise de Fenômenos Biomédicos da Universidade do Estado do Amazonas, Universidade do Estado do Amazonas, Manaus, Brazil; ^4^ Departamento de Morfologia, Universidade Federal do Amazonas, Manaus, Brazil; ^5^ Departamento de Saúde Coletiva, Universidade Federal do Amazonas, Manaus, Brazil; ^6^ Faculdade de Farmácia, Universidade Nilton Lins, Manaus, Brazil; ^7^ Programa de Pós-Graduação em Medicina Tropical, Universidade do Estado do Amazonas, Manaus, Brazil; ^8^ Departamento de Genética, Evolução, Microbiologia e Imunologia, Universidade Estadual de Campinas, Campinas, Brazil; ^9^ Diretoria de Ensino e Pesquisa, Fundação Hospitalar de Hematologia e Hemoterapia do Amazonas, Manaus, Brazil; ^10^ Programa de Pós-Graduação em Imunologia Básica, Instituto de Ciências Biológicas, Universidade Federal do Amazonas, Manaus, Brazil

**Keywords:** gametocyte, ookinete, malaria, transmission-blocking, antimalarial drug

## Abstract

Methylene blue (MB) is an alternative for combating drug-resistant malaria parasites. Its transmission-blocking potential has been demonstrated *in vivo* in murine models, *in vitro*, and in clinical trials. MB shows high efficacy against *Plasmodium vivax* asexual stages; however, its efficacy in sexual stages is unknown. In this study, we evaluated the potential of MB against asexual and sexual forms of *P. vivax* isolated from the blood of patients residing in the Brazilian Amazon. An *ex vivo* schizont maturation assay, zygote to ookinete transformation assay, direct membrane feed assay (DMFA), and standard membrane feed assay (SMFA) using *P. vivax* gametocytes with MB exposure were performed. A cytotoxicity assay was also performed on freshly collected peripheral blood mononuclear cells (PBMCs) and the hepatocyte carcinoma cell line HepG2. MB inhibited the *P. vivax* schizont maturation and demonstrated an IC_50_ lower than that of chloroquine (control drug). In the sexual forms, the MB demonstrated a high level of inhibition in the transformation of the zygotes into ookinetes. In the DMFA, MB did not considerably affect the infection rate and showed low inhibition, but it demonstrated a slight decrease in the infection intensity in all tested concentrations. In contrast, in the SMFA, MB was able to completely block the transmission at the highest concentration (20 µM). MB demonstrated low cytotoxicity to fresh PBMCs but demonstrated higher cytotoxicity to the hepatocyte carcinoma cell line HepG2. These results show that MB may be a potential drug for vivax malaria treatment.

## 1 Introduction

Malaria is a critical global parasitic disease and it was responsible for about 247 million cases and 619,000 deaths globally in 2021. *Plasmodium falciparum* is still the most prevalent malaria parasite in the African, Eastern Mediterranean, and Western Pacific Regions though *P. vivax* is the predominant parasite in the Americas (World Health Organization, 2022). Different studies across the globe have shown that some parasites are resistant to the antimalarials currently available (Gupta et al., 2015), especially chloroquine (CQ), which is the first-line treatment for vivax malaria in the Americas (Garavelli and Corti, 1992; Baird, 2009; Kittichai et al., 2019; World Health Organization, 2022).

Faced with drug resistance, new drugs must be investigated or the current ones repurposed (Lover et al., 2018; Su et al., 2019). First synthesized in 1876, methylene blue (MB) was in use as an antimalarial until the 1950s (Guttmann and Ehrlich, 1891; Schirmer et al., 2003) and was used for the treatment of a range of diseases (Schirmer et al., 2003). Currently, MB has been suggested for use in the treatment of COVID-19 due to its ability to inhibit the excessive production of reactive oxygen species and cytokines (Scigliano and Scigliano, 2021).

The antiplasmodial activities of MB have been evaluated in both pre-clinical and clinical trials against several *Plasmodium* species, including strains that are resistant to standard antimalarials (Pascual et al., 2011). Against *P. falciparum*, MB has shown itself to be active against both asexual and sexual stages (Adjalley et al., 2011; Pascual et al., 2011; Okombo et al., 2012), thus showing its potential use for blocking transmission (Lu et al., 2018; Müller et al., 2019). Moreover, MB appears to be safe in glucose-6-phosphate dehydrogenase (G6PD) deficient patients with slightly reduced hemoglobin values (Müller et al., 2013). Against *P. vivax* parasites, studies on MB have illustrated its high efficacy against the asexual stages (Suwanarusk et al., 2015; Wirjanata et al., 2015); however, its effect on the sexual stages and consequent blockage of transmission is not yet known. Accordingly, herein, we investigated the antimalarial activity of MB against *P. vivax* isolates from the Brazilian Amazon *via ex vivo* assays on asexual and sexual maturation stages (ring to schizont and zygote to ookinete, respectively) and its transmission-blocking effect *via* direct membrane feeding assays (DMFA) and standard membrane feeding assays (SMFA).

## 2 Materials and methods

### 2.1 Methylene blue compound

The compound methylene blue (Sigma-Aldrich) was diluted in Milli-Q^®^ water to make a stock solution of 5 mg/mL, which was maintained at -80°C.

### 2.2 Ethics statement and sample collection

All patients with *P. vivax* infection included in the project gave written informed consent. All protocols were approved by the ethical review board of Fundação de Medicina Tropical Doutor Heitor Viera Dourado (FMT-HVD) (CAAE: 50812815.0.0000.0005, approval number 1.358.054). A 9 mL sample of *P*. *vivax*-infected blood was withdrawn by venipuncture and placed in heparinized tubes. The patients were recruited at FMT-HVD, Manaus, Amazonas, and the following inclusion criteria were applied: over 18 years of age, a positive microscopy diagnosis for *P. vivax* mono-infection, parasitemia higher than 1,000 parasites/µl, and absence of any antimalarial treatment in the last 60 days. After sample collection, all patients were treated according to the Brazilian Malaria Treatment Guidelines (Brasil, 2021).

A 4 mL blood sample from three healthy adult blood donors was collected *via* venipuncture in EDTA tubes in order to obtain PBMCs for cell viability assay. All donors gave written informed consent and protocols were approved by the ethical review board of Fundação Hospitalar de Hematologia e Hemoterapia do Amazonas (CAEE: 51257921.2.0000.0009, approval number #4.982.395/2021).

### 2.3 *Ex vivo* activity against asexual *P. vivax* isolates

The *ex vivo* susceptibility of *P. vivax* isolates to MB was evaluated *via* a modified schizont maturation assay (Russell et al., 2003). Only blood samples with over 50% ring stage were used. The blood samples were centrifuged (400 x g, 5 min, and 25 °C), and the resulting red blood cell pellet was washed in RPMI-1640 medium (Sigma-Aldrich) and filtered through cellulose columns (Sigma-Aldrich) for leukocyte removal. The parasitized erythrocyte suspension was then washed with RPMI 1640 three times. The pellet was resuspended in IMDM medium (Sigma-Aldrich) supplemented with 20% AB serum (Sigma) to 2% final hematocrit. The parasitized cells were distributed in 96-well plates that were preloaded with ten concentrations in multiples of two MB (0.1 to 51.2 nM) or chloroquine (CQ) as the positive control (1.95 to 1,000 nM), as well as the vehicle as a negative control. The cultures were thereafter incubated at 37°C for 36 h to 48 h in a hypoxia chamber (with 5% CO_2_, 5% O_2_, and 90% N_2_ gas mixture). Schizont count per 200 parasites was determined by microscopic examination and the treated wells were compared to those of the control wells and the IC_50_ values were determined. The IC_50_ of MB was evaluated in ten different *P. vivax* isolates (biological replicates).

### 2.4 Production of *P. vivax ex vivo* ookinetes and inhibition assay

To check the ability of MB to inhibit the conversion of zygotes into ookinetes, the production of *P. vivax* ookinetes was performed as previously described (Fabbri et al., 2021). Leukocyte-depleted parasitized blood was resuspended in an exflagellation medium (10 mM Tris, 170 mM NaCl, 10 mM glucose, 25 mM NaHCO_3_, 50 to 100 mM xanthenuric acid, and 20% fetal calf serum, pH 8.4) to 20% hematocrit and incubated for one hour at 25 °C with ambient gas conditions. After the first incubation, for the separation of the sexually mature stages of the parasite (zygotes), the blood suspension was centrifuged (400 x g, 5 min, and 36 °C), and the pellet was resuspended with RPMI 1640 medium before purification with 45% Percoll (GE Healthcare). The resultant pellet was washed and resuspended in ookinete medium (IMDM medium, 50 mg/L of hypoxanthine, and 20% heat-inactivated AB human serum, pH 8.0) to 20% final hematocrit. The cultures were incubated with MB at a final concentration of 10 μM and without the drug (control group) at 25 °C for 24 hours with ambient gas conditions to enable ookinete development. The number of ookinetes per 1,000 erythrocytes was determined using an optical microscope and the drug inhibition percentage was calculated. In this assay, three different *P. vivax* isolates (biological replicates) were evaluated in duplicate.

### 2.5 *Anopheles aquasalis* colony

*Anopheles aquasalis* mosquitoes were obtained from a well-established colony at the insectary facility of the Medical Entomology laboratory at FMT-HVD, Manaus, Amazonas, Brazil, as per established protocols (Pimenta et al., 2015). The colonies were maintained at a constant temperature (24 to 26°C) and relative humidity (70 to 80%). Mosquito larvae were hatched in water at room temperature and fed with commercial fish food (TetraMin^®^). The larvae developed into pupa and then matured into adult mosquitoes, which were maintained with water and 10% sucrose solution before their use in the experiments. Adult females of *An. Aquasalis* aged 3 to 6 days old and deprived of the 10% sucrose solution 24 h before each assay were used in the subsequent experiments.

### 2.6 *P. vivax* direct membrane feeding assay

The DMFA was performed as previously described by Fabbri et al. (2021). *P. vivax-*infected blood was centrifuged to remove plasma (400 g, 5 min, and 36 °C). In a laminar flow chamber with the temperature controlled at 32 to 37 °C, the erythrocytes were washed twice in RPMI-1640 before reconstitution with inactivated AB human serum (Sigma-Aldrich) to 40% final hematocrit. One milliliter of this blood solution (400 μL of erythrocytes pellet + 600 μL inactivated AB human serum) was offered to five groups of mosquitoes *via* membrane feeding devices at 37 °C with each one containing 120 to 150 females. During the DMFA assay, the MB concentrations were added directly to the blood solution at the beginning of mosquito feeding, which lasted from 30 to 120 min. Two independent experiments were performed: the first with MB concentrations (5 µM and 10 µM) and a drug-free control (Control A), and the second with an MB concentration of 20 µM and a drug-free control (Control B). After feeding, only fully engorged females were transferred to a new cage and maintained in the infected-mosquito room (between 24 to 28 °C and 70 to 80% humidity) with a 10% sucrose solution *ad libitum*. At seven days post-infection, the mortality rate was determined and the surviving mosquitoes from each experimental group were dissected. The midguts were stained with 2% mercurochrome stain (Sigma-Aldrich) for 10 minutes. The mosquito infection rate (% of mosquitoes with the presence of 1 or more oocysts in the midgut) and infection intensity (mean number of oocysts per midgut infected) were determined. This assay was conducted in five different *P. vivax* isolates (thus 5 biological replicates per test group).

### 2.7 *P. vivax* standard membrane feeding assay

In order to determine whether MB possesses a gametocytocidal action, the SMFA methodology was performed using a parasite culture pre-exposed to this drug. Parasite maintenance was carried out as described previously (Ramos et al., 2021). After leukocyte depletion, for each MB concentration (5, 10, and 20 μM) and control group (without drug), 400 µl of the pellet was cultured in 25 cm^2^ bottles using IMDM supplemented with 20% inactive AB human serum to a final hematocrit of 2%. Parasites were incubated *ex vivo* under gas conditions of 5% CO_2_, 5% O_2_, and 90% N_2_ and at a temperature of 37°C for 6 h. The samples obtained from the culture were centrifuged and the media was removed. The pellet was resuspended in RPMI at a proportion of 1:1 and centrifuged (400 g, 5 min, and 36 °C) for washing of the MB compound (washout). The cell pellet was resuspended to a 40% final hematocrit with inactive AB human serum (Sigma-Aldrich) and the blood solution (as described in section 2.6) was offered to the mosquitoes for a maximum of 120 minutes. At seven days post-infection, each experimental group was dissected as previously described in the DMFA assay. This assay was conducted with five different *P. vivax* isolates (thus 5 biological replicates per test group). **Table 1** of the **Supplementary Material** summarizes the three methods used in this study to verify the efficacy of MB against sexual forms of *P. vivax*.

### 2.8 Methylene blue cytotoxicity assay

#### 2.8.1 Peripheral blood mononuclear cells

To check the PBMCs viability, the alamarBlue™ (7-Hydroxy-3H-phenoxazin-3-one-10-oxide sodium salt) method was performed as previously described with few modifications (Avila-Alejo et al., 2017; Kumar et al., 2018). PBMCs were isolated using a density gradient medium using Ficoll-Paque™. Four milliliters of blood from three healthy adults were centrifuged and the plasma was removed. The erythrocyte pellet was diluted in PBS with 10% fetal fovine ferum (FBS) and was added to Ficoll-Paque™. After centrifugation (450 x g, 30 min, and 20 °C), the white blood interface was washed in PBS 1x (200 x g, 15 min, and 20 °C) and the cell pellet was resuspended in IMDM medium supplemented with 10% FBS and 50 μg/mL gentamicin to a concentration of 500,000 cells/mL. A measurement of 200 µL of this solution was added to a 96-well plate and incubated for 24 h (37 °C and 5% CO_2_). Then, the MB was added at 5-serial dilution concentrations (1.25 to 20 µM) and incubated for 24, 48, or 72 h (37 °C and 5% CO_2_). At these time points, alamarBlue™ (Sigma-Aldrich) was added at 0.4% concentration and incubated for 2 h (37 °C and 5% CO_2_). The fluorescence reading was performed on the GloMax^®^ Explorer plate reader (Promega) using excitation and emission filters of 520 nm and 580 to 640 nm (green), respectively. The drug concentrations and controls were evaluated in duplicate, and the percentage of cell viability was expressed as a percentage relative to untreated control (cells with a medium plus vehicle without MB).

#### 2.8.2 Human hepatocarcinoma cell line

For cell viability in the human hepatocarcinoma cell line (HepG2), the 3-[4,5-dimethyl-thiazol-2-yl]-2,5-diphenyltetrazolium bromide (MTT) assay was performed as previously described (Kumar et al., 2018a). Briefly, the cells were cultured in Dulbecco’s modified Eagle medium (DMEM, Sigma), supplemented with 10% FBS, and kept in an incubator at 37 °C and 5% CO_2_. Cells were plated in a 96-well plate at a concentration of 10,000 cells/well and incubated for 16 h to 70% confluence. The MB was then added at 7-serial dilution concentrations (0.14 to 100 µM) and incubated for 24, 48, or 72 h (37 °C and 5% CO_2_). Absorbance reading was performed on a CLARIOstar plate reader (BGMtech) at a wavelength of 570 nm (OD570). The drug concentrations and controls were evaluated in triplicate. The percentage of cell viability was expressed as a percentage relative to the untreated control, and the EC_50_ was calculated.

### 2.9 Statistical analysis

The IC_50_ was analyzed with a normalized response-variable slope. The EC_50_ was determined by nonlinear regression using a three-parameter dose-response curve. Normality was evaluated using a Shapiro-Wilk or Kolmogorov-Smirnov test. The comparison between the two groups was performed using an unpaired t-test or Mann-Whitney test. Comparison among multiple groups was performed using one-way ANOVA with Tukey’s multiple comparisons test or the Kruskal-Wallis test followed by Dunn’s multiple comparisons post-test. Statistical significance was defined as p < 0.05. All statistical analyses were conducted using GraphPad Prism (version 9) software (GraphPad Software Inc., San Diego, CA).

## 3 Results

### 3.1 Methylene blue inhhibit *P. vivax* asexual stages *ex vivo* maturation presenting an IC50 lower than chloroquine

To check the ability of MB to inhibit the ring to schizont maturation, ten *P. vivax* isolates (**Table 1**) were used in the schizont maturation assay (SMA). The IC_50_ of both drugs (MB and CQ) was determined for each isolate. The median CQ (control drug) and MB IC_50_ were 28.14 ± 70.8 (range from 7.97 to 243.2) and 2.41 ± 5.36 (range from 0.01 to 18.52) nM, respectively. MB demonstrated an inhibitory effect on *P. vivax* maturation in the asexual stage (p-value = 0.0001). MB IC_50_ was 11.68-fold lower than the control drug.

**Table 1:** *Ex vivo* activity against asexual *P. vivax* per isolate.

P. vivax isolates (n)	Parasitemia (μL)	CQ IC_50_ (nM)	MB IC_50_ (nM)
**1**	4,371	12.43	18.52
**2**	8,040	10.43	1.34
**3**	3,375	72.69	0.01
**4**	10,230	38.83	2.41
**5**	6,013	29.3	1.14
**6**	9,548	7.97	3.95
**7**	13,590	243.2	1.37
**8**	6,360	45.73	5.90
**9**	22,080	12.18	2.90
**10**	7,560	26.97	2.40
**Median± SD**	**-**	28.14 ± 70.80	2.41 ± 5.36

A total of ten isolates were used to evaluate the ability of MB to inhibit the maturation from ring to schizont. The peripheral parasitemia was calculated by the number of parasites per 200 leucocytes in relation to the patient’s white blood cells per microliter of blood. CQ – Chloroquine; MB – Methylene blue. The IC_50_ for each isolate was analyzed using a normalized response-variable slope.

### 3.2 Methylene blue impairs zygote to ookinete transformation *ex vivo*

**Figure 1:**
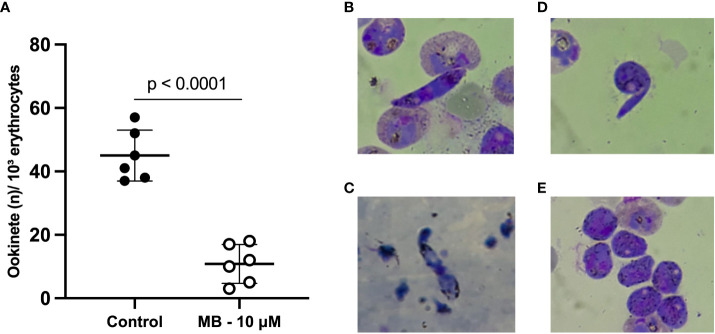
Methylene blue activity in the ex vivo Plasmodium vivax ookinete assay. Mean number of ookinetes per 10³ erythrocytes found in stained thin smears from three independent assays (isolates) tested in duplicate after 24 hours of P. vivax zygote culture at 25°C in the presence or absence (control group) of methylene blue at 10 µM. Each circle represents one replicate **(A)**. Photos show thin smears stained with Panoptic staining kit after 24 hours of P. vivax zygote culture at 24°C. **(B, C)** demonstrate mature ookinetes in the absence of MB (control group). **(D, E)** demonstrate only immature sexual forms – zygotes in the presence of MB at 10 µM. P-value (p < 0.0001) was calculated using the unpaired t test.

The capacity of MB to block zygote to ookinete transformation was evaluated in three *P. vivax* clinical isolates (evaluated in duplicates) (**Figure 1A**). The mean number of ookinetes per 10³ erythrocytes in MB at 10 µM was 10.83 ± 6.11 compared to 45.0 ± 8.02 in the control group (p*-*value = 0.001). The inhibition of ookinete transformation reached a mean of 75.9 ± 13.9% in the MB-treated group. The MB treatment impaired the maturation of *P. vivax* ookinetes (control) since mature ookinetes were evidenced in the control group (**Figures 1B, C**), but in the presence of MB, only immature forms (round and stem forms) were observed (**Figures 1D, E**).

### 3.3 Methylene blue blocks transmission only with drug pre-exposure of gametocytes

**Figure 2:**
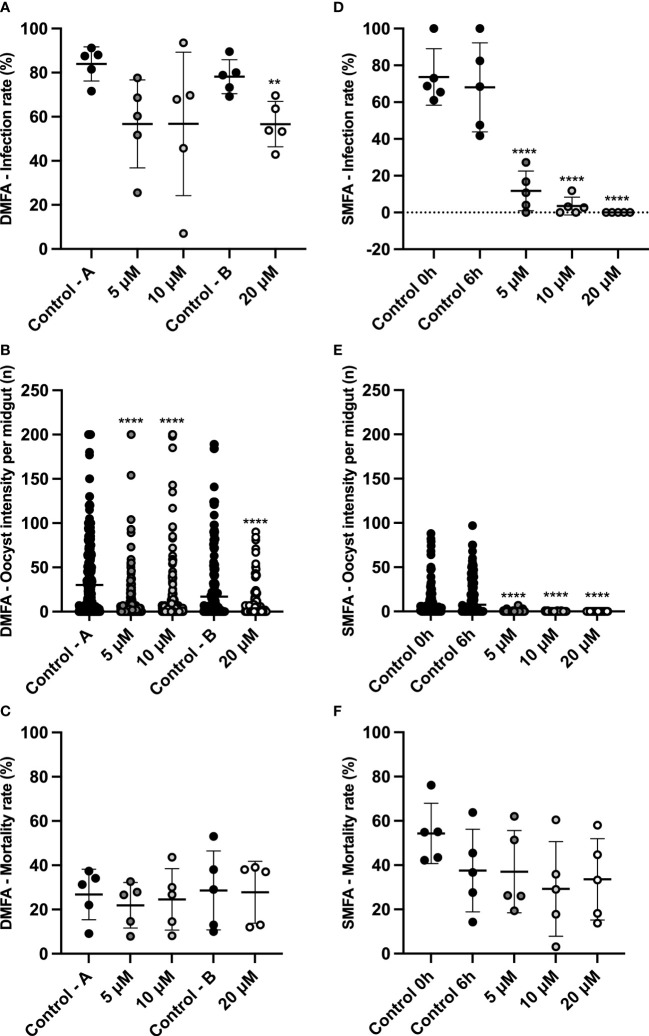
MB transmission-blocking activity for *Plasmodium vivax* using DMFA and SMFA. DMFA infection rate (% of mosquitoes with the presence of 1 or more oocyst in the midgut)) **(A)**. DMFA intensity (mean oocysts per infected midgut) **(B)**. DMFA mortality (mean number of dead mosquitoes at seven days post-infection) **(C)**. SMFA infection rate (% of mosquitoes with the presence of 1 or more oocysts in the midgut) **(D)**. SMFA intensity (mean of oocysts per infected midgut) **(E)**. SMFA mortality (mean number of dead mosquitoes seven days post-infection) **(F)**. Both assays were evaluated in five independent experiments (isolates) by feeding *An. aquasalis* with blood without MB (Control A, B, 0 h, and 6 h) or blood with different concentrations of MB (5, 10, and 20 µM). DMFA infection rate p*-*values were calculated using one-way ANOVA with Tukey’s multiple comparisons tests (for Control A, 5 μM, and 10 μM concentrations) and the unpaired t-test (for control B and 20 μM concentration). DMFA infection intensity p-values were calculated using the Kruskal-Wallis test followed by Dunn’s multiple comparison post-test (for Control A, 5 μM, and 10 μM concentrations) and the Mann-Whitney test (for control B and 20 μM concentration). SMFA infection rate p-values were calculated using one-way ANOVA with Tukey’s multiple comparisons tests, and infection intensity was calculated using the Kruskal-Wallis test followed by Dunn’s multiple comparisons post-test. Asterisks (**) and (****) represent significant differences (p <0.01 and p <0.0001, respectively) in relation to control A or control B (for DMFA) and control 6 h (for SMFA).

The ability of MB to block the transmission of viable gametocytes was evaluated using DMFAs and SMFAs. In each assay, five *P. vivax* isolates were evaluated (**Supplementary Material Table 2**). The infection rate, intensity, and mortality are described in **Figure 2**. Although in the DMFA assay, a significant difference was demonstrated between control B and the highest concentration of MB (**Figure 2A**); all tested concentrations showed low inhibition (5 μM – 32.4%, 10 μM – 32.4%, and 20 μM – 27.6%). However, MB was able to affect the oocyst intensity in all the tested concentrations (**Figure 2B**).

In the SMFA assay, using blood with gametocytes pre-exposed to MB for six hours (SMFA assay), the infection rate and intensity were extremely reduced (**Figures 2D, E**), thus showing high inhibition in all tested concentrations (5 μM – 82.7%, 10 μM – 94.8%, and 20 μM – 100.0%) with a total blocking of transmission at the highest concentration. In addition, the IC_50_ value (mean of 3.4 ± 1.1 μM) was calculated, and it demonstrated that MB is effective at low concentrations. None of the MB concentrations tested interfered with the *An. aquasalis* mortality rate in either of the membrane assay methods (**Figures 2C, F**). **Table 2** shows the data for the infection and intensity rate from the DMFA and SMFA assays.

**Table 2:** Infection and intensity rates of DMFA and SMFA assay.

Methodology	MB compound	Infection rate - % (Mosquitoes examined)	Infection intensity (mean ± SD)
**DMFA**	**Control A**	84.0 (233)	54.08 ± 48.47
	**5 μM**	56.7 (218)	22.56 ± 36.52^a^
	**10 μM**	56.8 (209)	30.19 ± 46.53^a^
	**Control B**	78.2 (124)	43.71 ± 46.47
	**20 μM**	56.6 ^b^ (114)	20.03 ± 22.77^a^
**SMFA**	**Control 0 h**	72.0 (100)	12.3 ± 19.9
	**Control 6 h**	65.7 (99)	17.6 ± 21.9
	**5 μM**	8.4^a^ (119)	0.17 ± 0.77^a^
	**10 μM**	2.8 ^a^ (141)	0.03 ± 0.2^a^
	**20 μM**	0.0^a^ (112)	0.0 ± 0.0^a^

Infection rate (% of mosquitoes with the presence of 1 or more oocysts in the midgut) and infection intensity (mean of oocysts per infected midgut) from five independent assays (isolates) of each methodology were determined. DMFA infection rate p-values were calculated using one-way ANOVA with Tukey’s multiple comparisons tests (for Control A, 5 μM, and 10 μM concentrations) and an unpaired t-test (for control B and 20 μM concentration). DMFA infection intensity p-values were calculated using the Kruskal-Wallis test followed by Dunn’s multiple comparison post-test (for Control A, 5 μM, and 10 μM concentrations) and the Mann-Whitney test (for control B and 20 μM concentration). SMFA infection rate p-values were calculated using one-way ANOVA with Tukey’s multiple comparisons tests, and infection intensity was calculated using the Kruskal-Wallis test followed by Dunn’s multiple comparisons post-test. a: p-value <0.0001; b: p-value <0.01 when compared to control A or control B (for DMFA) and control 6 h (for SMFA). Results are the mean of five independent biological replicates.

### 3.4 Methylene blue demonstrated low cytotoxicity to PBMCs but higher to the hepatocarcinoma cell line HepG2

**Figure 3:**
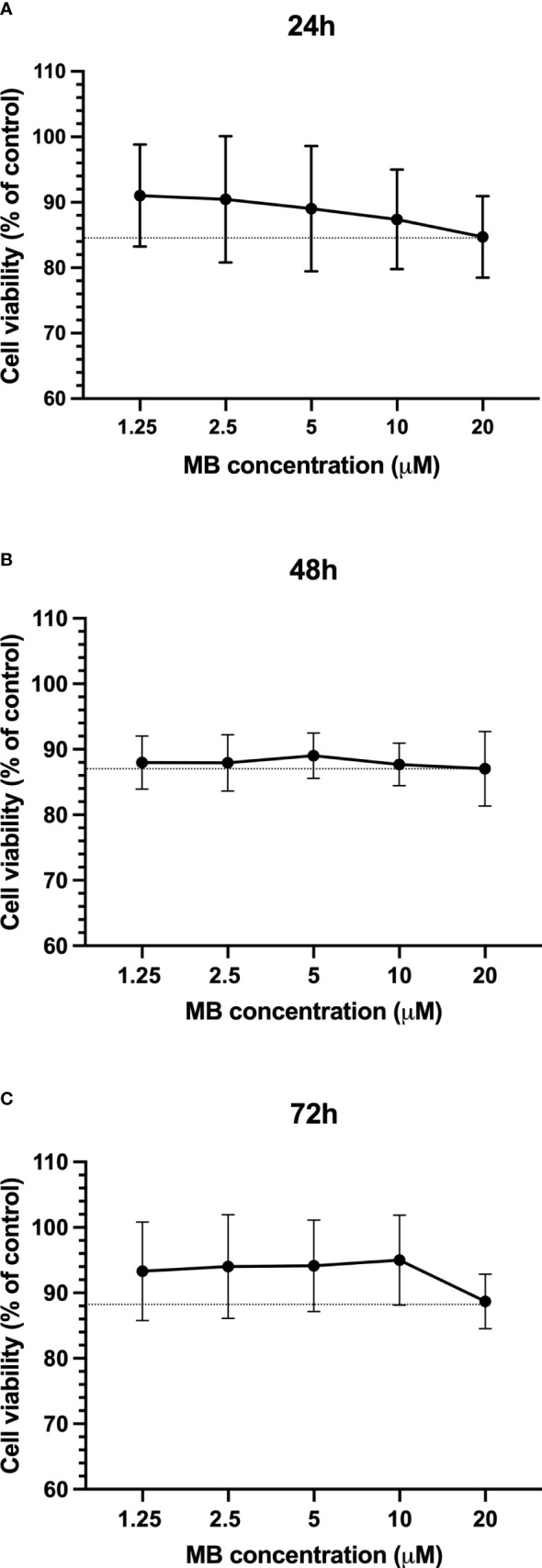
MB cytotoxicity on PBMCs. Cell viability of PBMCs presented as a percentage of control after 24h **(A)**, 48h **(B)**, and 72h **(C)** of exposure to different concentrations of MB. The data represent the mean values of three experiments (± SD). The dotted line represents the minimum percentage of cell viability in each exposure time.

In order to evaluate whether the MB concentrations used in the assays are cytotoxic, cell viability assays were carried out. After the isolation of PBMCs from the blood of three healthy adult donors, the cells were exposed to five concentrations of MB. The reading was performed at three time points: 24 , 48 , and 72 h. As shown in **Figure 3**, cell viability was reduced by 12 to 15% relative to untreated controls. Specifically, at the highest concentration (20 µM), the mean percentage of cell viability after 24 h of exposure was 84.7%, 48 h was 87.0%, and 72 h was 83.9%. The reduction in cell viability between MB and untreated controls was not statistically significant (Kruskal-Wallis test) at the three time points evaluated. Moreover, no difference in the cell viability profile between the different exposure times was found.

Additionally, MB demonstrated a moderate citotoxicity profile against hepatocarcinoma cell line HepG2, yielding an EC_50_ of 2.68 ± 1.81 µM (**Supplementary Material Figure 1**).

## 4 Discussion

This study demonstrates the *ex vivo* and *in vivo* activity of methylene blue against different stages of *Plasmodium vivax* development. This activity against the *P. vivax* asexual erythrocytic stage has already been reported with isolates from Indonesia where CQ resistance is widespread (Wirjanata et al., 2015). Using the same methodology as previously described, in both studies, MB had an inhibitory concentration lower than that of CQ. Although CQ is still the standard and first-line treatment against vivax malaria in Brazil (Brasil, 2021; World Health Organization, 2021), the emergence of chloroquine-resistant strains of *P. vivax* has already been reported in Brazil and other countries (Gonçalves et al., 2014; Kittichai et al., 2019).

Regarding its activity against sexual stages, MB demonstrated high levels of inhibition in the *ex vivo* ookinete transformation assay. Likewise, it was possible to observe high levels of inhibition in the SMFA, including the complete blocking of transmission at the highest concentration. In contrast to these data, the experiments performed using the DMFA were not as satisfactory as those mentioned above. Although a decrease in the intensity of infection was observed, the infection rates of the MB groups were similar to the control groups.

This difference between the DMFA, the SMFA, and the ookinete transformation assay results may be explained by the pre-exposure of the *P. vivax* forms to the MB. In the DMFA assay, the *P. vivax* blood solution was not subjected to pre-exposure with MB since the drug solution was added directly into the membrane feeder device of each group at the time of feeding. The DMFA methodology is widely used when the parasitized blood source is obtained directly from a patient and when the experiment can be performed within a few hours (Miura et al., 2020). Since MB showed only slight inhibition in this methodology, which was different from our findings in the *ex vivo* ookinete transformation assay, we performed the SMFA in which the drug is incubated with the parasites, and, subsequently, these are used to carry out membrane feeding of the anophelines. The SMFA is widely used in experiments involving *P. falciparum* and is known as the gold standard for transmission-blocking assays (Churcher et al., 2012). The limitation in using this assay with *P. vivax* is the absence of a long-term culture that produces gametocytes to infect mosquitoes. Thus, we adapted the protocol used for *P. vivax*, and gametocytes were pre-exposed to MB culture for 6 h instead of 24 h, which is the usual period. The length of exposure was chosen based on our previous data, which showed that *P. vivax* gametocytes were viable, and infection of *Anopheles* remains unaltered when mosquitoes are fed with gametocytes of 6h culture compared with the same isolate without culture (Ramos et al., 2021).

Furthermore, it is important to mention that in all the methodologies used (DMFA, SMFA, and ookinete production), the plasma of the patients was removed and replaced by inactivated AB serum. This is a key step for understanding the intervention of the compound without interference by the host immune system

However, in the DMFA, the leukocytes are not removed, which may partially explain the low MB inhibition profile shown in this assay. The presence of leukocytes in the assay could interfere with parasite viability through phagocytosis or other immune mechanism and limit infection of the mosquitoes, and this, therefore, masks the effect of MB. Nevertheless, all performed assays have internal control, and even if the leukocytes present in the DMFA play a role in the infection of the mosquitoes, this effect is normalized by the control without drugs.

Therefore, we highlight that besides MB being effective in blocking transmission after 6 hours of incubation, evaluating its effect in the real scenario, which includes evaluating the MB potential beyond the immune system, is needed.

Accordingly, the period of gametocyte exposure to MB could be a key factor in the transmission-blocking potential of MB. This study suggests a cytotoxic action on gametocytes and corroborates the findings that have already been extensively demonstrated, i.e., that MB has gametocidal activity against mature *P. falciparum* gametocytes *in vitro* (Adjalley et al., 2011; Alessandro et al., 2013; Wadi et al., 2018) and *in vivo* (Coulibaly et al., 2009; Adjalley et al., 2011; Alessandro et al., 2013; Coulibaly et al., 2015; Dicko et al., 2018).

The mechanism of action of MB against *P. falciparum* is through its inhibition of the glutathione-dependent degradation of heme (Garavito et al., 2007). It also kills the parasite by increasing the intracellular concentration of oxidants and toxic products that are harmful to both the asexual and gametocyte stages (Coulibaly et al., 2009; Lu et al., 2018; Wadi et al., 2018). However, even though our study suggests a cytotoxic action in the gametocytes, it is important to emphasize that the specific mechanism of action of MB for *P. vivax* remains unknown.

The *in vitro* PBMC cytotoxicity assays using the same MB concentrations evaluated in the antimalarial assays suggest that MB is likely to be safe for at least up to 20 µM. However, our study also showed moderate MB cytotoxicity in the hepatocarcinoma cell line HepG2. Similarly, a prior study has shown that MB is more toxic in tumor cell lines, presenting EC_50_ 20,000 folds higher in normal PBMCs compared to tumor cell lines (Kirszberg et al., 2005). Despite the MB in this study presented an EC_50_ of 11 µM (3,530 µg/mL) in PBMCs, only a slight reduction in cell viability was found at 20 µM. Unfortunately, it was not possible to determine the EC_50_ for PBMCs since the toxicity was minimum in all concentrations tested.

Notably, MB was previously used for malaria treatment in the late 19th and 20th century, and a systematic review on MB safety and efficacy has evaluated 21 studies, collecting data from 1,504 malaria patients treated with MB, and found only mild urogenital and gastrointestinal effects and urine with a blue coloration (Lu et al., 2018). Furthermore, five clinical studies with G6PD-deficient children in West Africa have demonstrated that MB is well tolerated but requires clinical attention since a slight reduction in hemoglobin was detected (Coulibaly et al., 2009; Müller et al., 2013; Coulibaly et al., 2015 Dicko et al., 2018; Jorge et al, 2019).

Therefore, MB should be considered as an alternative drug in the treatment of malaria, especially in the context of the emergence and spread of drug-resistant *Plasmodium* species and strains across several countries (Marques et al., 2014; Kittichai et al., 2019). Moreover, the development of new molecules is an expensive and time-consuming process, thus the recovery of old drugs is a strategy for an efficient and low-cost solution.

## Data availability statement

The original contributions presented in the study are included in the article/**Supplementary Material**. Further inquiries can be directed to the corresponding authors.

## Ethics statement

The studies involving human participants were reviewed and approved by Fundação de Medicina Tropical Doutor Heitor Viera Dourado (FMT-HVD) ethical board committee (CAAE: 50812815.0.0000.0005, approval number 1.358.054) Fundação Hospitalar de Hematologia e Hemoterapia do Amazonas (CAEE: 51257921.2.0000.0009, approval number #4.982.395/2021). The patients/participants provided their written informed consent to participate in this study.

## Author contributions

CF wrote the original draft, performed project administration, and carried out the experiments and formal data analyses. GR reviewed the manuscript and supported the SMFA methodology. DBS reviewed the manuscript and aided in carrying out the DMFA methodology. AT aided in carrying out the ookinete methodology for *P. vivax*. CF, LA, JN, IB, and AC coordinated and performed the cytotoxicity assays. ML was responsible for reviewing the final manuscript and aided in funding acquisition. WM and FC were responsible for reviewing the final manuscript. SL was responsible for the supervision, project administration, formal analysis, data curation, validation, review, and editing of the final manuscript. All authors read and approved the final version of the manuscript.
