# Supplementary material for: The activity of methylene blue against asexual and sexual stages of Plasmodium vivax
Source: Front Cell Infect Microbiol. 2023 Apr 18;13:1108366. doi: 10.3389/fcimb.2023.1108366 (PMC10152470; doi:10.3389/fcimb.2023.1108366)

Supplementary Material

The activity of methylene blue against asexual and sexual stages of *Plasmodium vivax*

Camila Fabbri*, Glenda Quaresma Ramos, Djane Clarys Baia-da-Silva, Alexandre Oliveira Trindade, Luis Carlos Salazar Alvarez, Juliana Costa Ferreira Neves, Ivanildes Santos Bastos, Allyson Guimarães Costa, Marcus Vinicius Guimarães Lacerda, Wuelton Marcelo Monteiro, Fabio Trindade Maranhão Costa, Stefanie Costa Pinto Lopes*

***Correspondence:**
Camila Fabbri
milafabbri@hotmail.com

Stefanie Costa Pinto Lopes
stefaniecplopes@gmail.com

Supplementary Material Table 1: The difference among the three methodologies performed to verify the efficacy of MB against *P. vivax* sexual forms

	**Ookinete assay**	**DMFA**	**SMFA**
**Purpose**	To verify the ability of MB to inhibit the transition from zygotes to ookinetes	To verify the ability of MB to block the transmission to *An. Aquasalis* by checking the presence of oocyts in the midgut after seven days post-infection	
**Type of assay**	*Ex vivo*	*In vivo*	*In vivo and ex vivo*
**Pre-exposure to MB?**	Yes, 24 hours, which is the period of transition from zygote to ookinete	No	Yes, six hours, the period for which *P. vivax* gametocytes are viable in culture according to Ramos et al. (2021)
**Target MB sexual form**	Zygote to ookinete	Gametocyte, zygote and ookinete	Gametocyte
**MB concentrations tested**	10 µM	5, 10 and 20 µM	

Supplementary Material Table 2: Infection and intensity rates of DMFA and SMFA assay per *P. vivax* isolates. Infection rate (% of mosquitoes with the presence of 1 or more oocyst in the midgut), infection intensity (mean oocysts per infected midgut) from five independent assays (isolates) for each methodology.

**DMFA**										
	**Control A**		**5 μM**		**10 μM**		**Control B**		**20 μM**	
**Assays**	**Infection rate (%)**	**Infection intensity (mean ± SD)**	**Infection rate (%)**	**Infection intensity (mean ± SD)**	**Infection rate (%)**	**Infection intensity (mean ± SD)**	**Infection rate (%)**	**Infection intensity (mean ± SD)**	**Infection rate (%)**	**Infection intensity (mean ± SD)**
1	91.2 (52/57)	70.53 ± 59.53	77.6 (38/49)	34.29 ± 51.93	93.5 (29/31)	75.42 ± 65. 93	73.3 (22/30)	44.9 ± 53.8	69.6 (16/23)	6.1 ± 11.2
2	71.6 (48/67)	31.36 ± 43.17	60.3 (35/58)	5.40 ± 8.63	67.9 (36/53)	4.96 ± 5.82	80.0 (24/30)	6.1 ± 11.2	63.6 (14/22)	11.1 ± 15.6
3	88.0 (44/50)	37.70 ± 29.53	25.5 (12/47)	0.85 ± 2.75	7.0 (4/57)	0.11 ± 0.45	78.9 (15/19)	30.3 ± 37.2	42.9 (12/28)	9.6 ± 23.1
4	81.5 (22/27)	26.74 ± 38.22	51.7 (15/29)	17.45 ± 26.72	69.7 (23/33)	16.36 ± 17.52	89.5 (17/19)	25.9 ± 32.4	53.8 (14/26)	13.4 ± 21.9
5	87.5 (28/32)	55.09 ± 51.52	68.6 (24/35)	7.40 ± 9.52	45.7 (16/35)	3.23 ± 6.04	69.2 (18/26)	14.4 ± 16.1	53.3 (8/15)	18.8 ± 23.9
**SMFA**										
	**Control (without culture)**		**Control (6 h culture)**		**5 μM (6 h culture)**		**10 μM (6 h culture)**		**20 μM (6 h culture)**	
**Assays**	**Infection rate (%)**	**Infection intensity (mean ± SD)**	**Infection rate (%)**	**Infection intensity (mean ± SD)**	**Infection rate (%)**	**Infection intensity (mean ± SD)**	**Infection rate (%)**	**Infection intensity (mean ± SD)**	**Infection rate (%)**	**Infection intensity (mean ± SD)**
1	61.1 (11/18)	12 ± 15.8	41.7 (10/24)	2.3 ± 6.6	27.3 (3/11)	1.2 ± 2.3	0.0 (0/22)	0.0 ± 0.0	0.0 (0/21)	0.0 ± 0.0
2	100.0 (14/14)	36.9 ± 29.3	82.4 (14/17)	11.8 ± 13.0	16.7 (3/18)	0.2 ± 0.4	11.8 (2/17)	0.12 ± 0.33	0.0 (0/27)	0.0 ± 0.0
3	68.8 (11/16)	3.6 ± 4.1	100.0 (18/18)	44.3 ± 17.6	4.0 (1/25)	0.04 ± 0.2	0.0 (0/34)	0.0 ± 0.0	0.0 (0/21)	0.0 ± 0.0
4	65.4 (17/26)	1.0 ± 1.8	47.6 (10/21)	12.3 ± 16.8	10.7 (3/28)	0.1 ± 0.3	2.7 (1/37)	0.03 ± 0.16	0.0 (0/18)	0.0 ± 0.0
5	73.1 (19/26)	15.3 ± 20.4	68.4 (13/19)	21.6 ± 26.3	0.0 (0/37)	0.0 ± 0.0	3.2 (1/31)	0.03 ± 0.18	0.0 (0/25)	0.0 ± 0.0

Supplementary Material Figure 1: MB cytotoxicity on HepG2 cells. Cell viability of HepG2 cells presented as percentage of control after 72h of exposure to different concentrations of MB. The data represent the mean values of three triplicates (± SD).

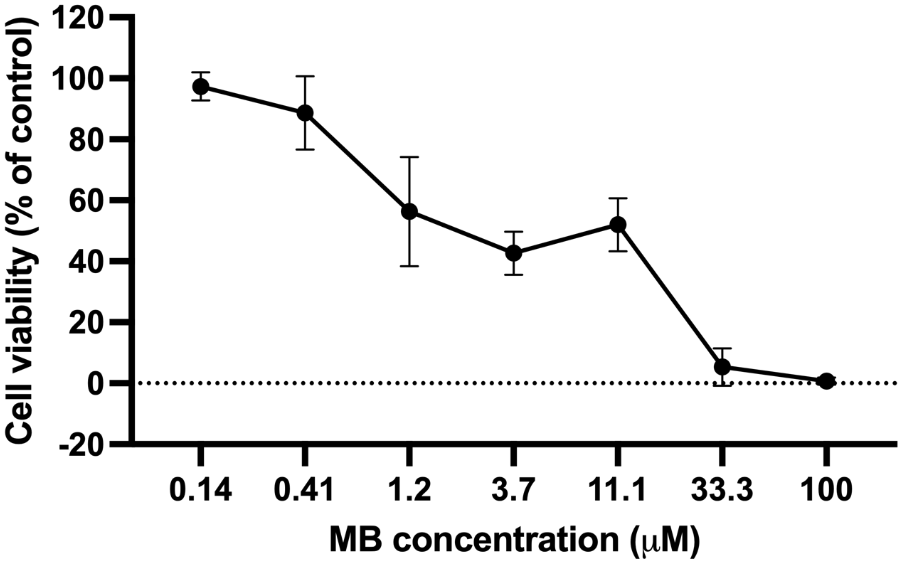

Supplement: Supplementary file 1 [file DataSheet_1.pdf]
